# Supplementary material for: Unveiling the Bioactive Potential of the Invasive Jellyfish Phyllorhiza punctata Through Integrative Transcriptomic and Proteomic Analyses
Source: Biomolecules. 2025 Aug 4;15(8):1121. doi: 10.3390/biom15081121 (PMC12383608; doi:10.3390/biom15081121)
Supplement: Supplementary file 1 [file biomolecules-15-01121-s001.zip › Suplementary Material/FileS3/PEPFOLD4/XP_065070690_8752/clusters.html]

 PEP-FOLD clusters


Download archive of all models  here   

|  |  |  |
| --- | --- | --- |
| # | file | sOPEP |
| # cluster 1 - Size: 6 |  |  |
|  | XP\_065070690\_8752-model1 | -26.4004 |
|  | XP\_065070690\_8752-model1.1 | -25.5208 |
|  | XP\_065070690\_8752-model1.2 | -25.4548 |
|  | XP\_065070690\_8752-model1.3 | -25.4127 |
|  | XP\_065070690\_8752-model1.4 | -25.2318 |
|  | XP\_065070690\_8752-model1.5 | -25.2155 |
| # cluster 2 - Size: 3 |  |  |
|  | XP\_065070690\_8752-model2 | -26.1613 |
|  | XP\_065070690\_8752-model2.1 | -26.1469 |
|  | XP\_065070690\_8752-model2.2 | -25.4548 |
| # cluster 3 - Size: 9 |  |  |
|  | XP\_065070690\_8752-model3 | -26.1222 |
|  | XP\_065070690\_8752-model3.1 | -25.8522 |
|  | XP\_065070690\_8752-model3.2 | -25.4025 |
|  | XP\_065070690\_8752-model3.3 | -25.3659 |
|  | XP\_065070690\_8752-model3.4 | -25.3475 |
|  | XP\_065070690\_8752-model3.5 | -25.2297 |
|  | XP\_065070690\_8752-model3.6 | -24.9417 |
|  | XP\_065070690\_8752-model3.7 | -24.8598 |
|  | XP\_065070690\_8752-model3.8 | -24.834 |
| # cluster 4 - Size: 4 |  |  |
|  | XP\_065070690\_8752-model4 | -25.7339 |
|  | XP\_065070690\_8752-model4.1 | -25.6435 |
|  | XP\_065070690\_8752-model4.2 | -25.5407 |
|  | XP\_065070690\_8752-model4.3 | -25.3345 |
| # cluster 5 - Size: 3 |  |  |
|  | XP\_065070690\_8752-model5 | -25.6647 |
|  | XP\_065070690\_8752-model5.1 | -25.5528 |
|  | XP\_065070690\_8752-model5.2 | -25.0463 |
| # cluster 6 - Size: 1 |  |  |
|  | XP\_065070690\_8752-model6 | -25.5618 |
| # cluster 7 - Size: 5 |  |  |
|  | XP\_065070690\_8752-model7 | -24.6263 |
|  | XP\_065070690\_8752-model7.1 | -23.7593 |
|  | XP\_065070690\_8752-model7.2 | -23.7184 |
|  | XP\_065070690\_8752-model7.3 | -23.6411 |
|  | XP\_065070690\_8752-model7.4 | -23.5846 |
| # cluster 8 - Size: 49 |  |  |
|  | XP\_065070690\_8752-model8 | -24.5782 |
|  | XP\_065070690\_8752-model8.1 | -24.4175 |
|  | XP\_065070690\_8752-model8.2 | -24.3319 |
|  | XP\_065070690\_8752-model8.3 | -24.2067 |
|  | XP\_065070690\_8752-model8.4 | -24.0386 |
|  | XP\_065070690\_8752-model8.5 | -23.9788 |
|  | XP\_065070690\_8752-model8.6 | -23.9716 |
|  | XP\_065070690\_8752-model8.7 | -23.94 |
|  | XP\_065070690\_8752-model8.8 | -23.918 |
|  | XP\_065070690\_8752-model8.9 | -23.9137 |
|  | XP\_065070690\_8752-model8.10 | -23.9063 |
|  | XP\_065070690\_8752-model8.11 | -23.8881 |
|  | XP\_065070690\_8752-model8.12 | -23.8873 |
|  | XP\_065070690\_8752-model8.13 | -23.8718 |
|  | XP\_065070690\_8752-model8.14 | -23.865 |
|  | XP\_065070690\_8752-model8.15 | -23.8574 |
|  | XP\_065070690\_8752-model8.16 | -23.824 |
|  | XP\_065070690\_8752-model8.17 | -23.7926 |
|  | XP\_065070690\_8752-model8.18 | -23.7901 |
|  | XP\_065070690\_8752-model8.19 | -23.7843 |
|  | XP\_065070690\_8752-model8.20 | -23.7704 |
|  | XP\_065070690\_8752-model8.21 | -23.7645 |
|  | XP\_065070690\_8752-model8.22 | -23.7596 |
|  | XP\_065070690\_8752-model8.23 | -23.7381 |
|  | XP\_065070690\_8752-model8.24 | -23.7296 |
|  | XP\_065070690\_8752-model8.25 | -23.7251 |
|  | XP\_065070690\_8752-model8.26 | -23.7245 |
|  | XP\_065070690\_8752-model8.27 | -23.7184 |
|  | XP\_065070690\_8752-model8.28 | -23.674 |
|  | XP\_065070690\_8752-model8.29 | -23.6627 |
|  | XP\_065070690\_8752-model8.30 | -23.6362 |
|  | XP\_065070690\_8752-model8.31 | -23.6303 |
|  | XP\_065070690\_8752-model8.32 | -23.6284 |
|  | XP\_065070690\_8752-model8.33 | -23.5334 |
|  | XP\_065070690\_8752-model8.34 | -23.5238 |
|  | XP\_065070690\_8752-model8.35 | -23.5009 |
|  | XP\_065070690\_8752-model8.36 | -23.4981 |
|  | XP\_065070690\_8752-model8.37 | -23.4534 |
|  | XP\_065070690\_8752-model8.38 | -23.4459 |
|  | XP\_065070690\_8752-model8.39 | -23.4335 |
|  | XP\_065070690\_8752-model8.40 | -23.3937 |
|  | XP\_065070690\_8752-model8.41 | -23.3794 |
|  | XP\_065070690\_8752-model8.42 | -23.3586 |
|  | XP\_065070690\_8752-model8.43 | -23.3433 |
|  | XP\_065070690\_8752-model8.44 | -23.3314 |
|  | XP\_065070690\_8752-model8.45 | -23.3049 |
|  | XP\_065070690\_8752-model8.46 | -23.2151 |
|  | XP\_065070690\_8752-model8.47 | -23.1569 |
|  | XP\_065070690\_8752-model8.48 | -22.808 |
| # cluster 9 - Size: 14 |  |  |
|  | XP\_065070690\_8752-model9 | -24.2399 |
|  | XP\_065070690\_8752-model9.1 | -24.1552 |
|  | XP\_065070690\_8752-model9.2 | -24.1203 |
|  | XP\_065070690\_8752-model9.3 | -24.1031 |
|  | XP\_065070690\_8752-model9.4 | -24.0792 |
|  | XP\_065070690\_8752-model9.5 | -24.0719 |
|  | XP\_065070690\_8752-model9.6 | -23.9094 |
|  | XP\_065070690\_8752-model9.7 | -23.8696 |
|  | XP\_065070690\_8752-model9.8 | -23.7245 |
|  | XP\_065070690\_8752-model9.9 | -23.5689 |
|  | XP\_065070690\_8752-model9.10 | -23.4223 |
|  | XP\_065070690\_8752-model9.11 | -23.3642 |
|  | XP\_065070690\_8752-model9.12 | -23.289 |
|  | XP\_065070690\_8752-model9.13 | -22.971 |
| # cluster 10 - Size: 6 |  |  |
|  | XP\_065070690\_8752-model10 | -24.2143 |
|  | XP\_065070690\_8752-model10.1 | -23.8671 |
|  | XP\_065070690\_8752-model10.2 | -23.8164 |
|  | XP\_065070690\_8752-model10.3 | -23.5855 |
|  | XP\_065070690\_8752-model10.4 | -23.5633 |
|  | XP\_065070690\_8752-model10.5 | -23.5161 |
|  |  |  |
